# Case Report: A Case of Epileptic Disorder Associated With a Novel *CNTN2* Frameshift Variant in Homozygosity due to Maternal Uniparental Disomy

**DOI:** 10.3389/fgene.2021.743833

**Published:** 2021-10-08

**Authors:** Wenjie Chen, Fei Chen, Yiping Shen, Zhixian Yang, Jiong Qin

**Affiliations:** ^1^ Department of Paediatrics, Peking University People’s Hospital, Beijing, China; ^2^ Department of Neurology, Guangzhou Women and Children's Medical Center, Guangzhou, China; ^3^ The Maternal and Child Health Care Hospital of Guangxi Zhuang Autonomous Region, Guangxi Birth Defects Prevention and Control Institute, Guangxi, China; ^4^ Department of Medical Genetics, Shanghai Children’s Medical Center, Shanghai Jiao Tong University School of Medicine, Shanghai, China; ^5^ Division of Genetics and Genomics, Boston Children’s Hospital, Boston, MA, United States; ^6^ Department of Neurology, Harvard Medical School, Boston, MA, United States; ^7^ Department of Pediatrics, Peking University First Hospital, Beijing, China

**Keywords:** *CNTN2*, epilepsy, uniparental disomy (UPD), neurodevelopmental disorder (NDD), genotype-phenotype correlation

## Abstract

**Background:** Contactin 2, encoded by *CNTN2* on chromosome 1q32.1, is a neural-specific glycoprotein and plays important roles in neurodevelopment. A deleterious homozygous variant in the *CNTN2* gene was previously reported to cause autosomal recessive cortical myoclonic tremor and epilepsy. Since then, there has been no further report confirming the association of *CNTN2* and epilepsy. Here, we reported one new case, who presented with epilepsy, carrying a novel homozygous frameshift variant in *CNTN2*. The clinical and genetic features of the patient were reviewed.

**Case presentation:** The male patient presented with preschool age-of-onset neurodevelopmental impairment and focal seizures of temporal origin, and responded to valproate. A trio-whole exome sequencing revealed a novel homozygous frameshift variant in *CNTN2* (c.2873_c.2874delCT, p.Thr958Thrfs). The patient’s mother was a heterozygous carrier while his father was wild-type; they were both unaffected and non-consanguineous. Further study revealed that maternal uniparental disomy (1q32.1) unmasked the heterozygous variant of *CNTN2* in the proband.

**Conclusions:** This case enhanced the gene–disease relationship between *CNTN2* and epilepsy, which will help to further understand this emerging disorder.

## Background

One deleterious homozygous mutation in the contactin two gene (*CNTN2*) was previously reported to be associated with epilepsy, familial adult myoclonic, 5 (FAME5, OMIM 615400) (Stogmann et al., 2013). The reported patients were from a consanguineous Egyptian family. He presented with early adolescence onset focal epilepsy which responded well to carbamazepine treatment. Myoclonus developed soon after seizure; he also presented with neuropsychiatric symptoms and borderline cognitive level. However, controversy exists about the diagnosis of “familial cortical myoclonic tremor with epilepsy” (FCMTE) that Stogmann et al. established in the report (Striano et al., 2013). Striano et al. have challenged about the nature of tremors that Stogmann et al. described as cortical origin. In addition, Striano et al. also inquired the difference of inheritance pattern presenting in the family compared with the previous patients with FCMTE (Striano et al., 2013).

Given the small number of mutations and lack of phenotypic information, the relationship between *CNTN2* and epileptic disorder is of limited support at this time. Reporting of additional case can further enhance the causal relationship and better understand the genotypic and phenotypic spectra. Herein, we presented a Chinese patient with cognitive and behavior deficit, neuropsychiatric disorders, and pediatric epilepsy of temporal origin, who carried a novel homozygous frameshift variant p.Thr958Thrfs in *CNTN2* gene. Moreover, we also reported an unusual inheritance of the *CNTN2* variant involving maternal uniparental disomy (UPD).

## Case Presentation

The 7-year-old boy was born at full term by cesarean section; his parents were healthy and non-consanguineous Chinese Han. The parents denied any family history of neurologic disease. The boy had his first attack of generalized tonic-clonic seizure at the age of 5 years. Seizures lasted for 4–5 min and 2–3 times per month at initiation.

Levetiracetam (LEV) was given 4 months later after the first seizure and seizure was free. However, 3 months later, seizure was recurrent and was refractory to control even with the highest dose of LEV (50 mg/kg/day). Since then, seizure became frequent and focal seizure types evolved including 1) attacks with presentation of fear or panic followed by a scared scream, hand holding and scratching, foot pedaling, then loss of awareness, followed by swallow and eye blink that lasted for 30–90 s, which occurred up to 6–7 attacks per day; and 2) pause in activity, gazing, then loss of awareness, followed by swallow or jerking of left facial muscle that lasted for 10 s to 1 min, several times a day. Oxcarbazepine (OXC, 30 mg/kg/day) was added 6 months later, yet it was still ineffective.

He had a history of development delay before seizure onset, mainly presenting in learning ability, and seizures had been exacerbating his poor academic performance based on the description of his parents. On examination at the age of 7 years, his cognitive function was impaired at mild-moderate level, including learning difficulties, memory deficits, hyperactivity, and attention deficit, and could not handle well with schoolwork. In addition, he also behaved aggressively and impulsively. The previous VEEG showed left temporal-PG1-frontal interictal epileptiform discharges, ictal suggesting temporal and frontal lobe origin. Brain MRI revealed no abnormality. We prescript valproate (VPA) to the patient and ordered a trio-exome sequencing.

## Sequencing Methods

Genomic DNA was extracted from peripheral blood samples of the proband and his parents. We planned a proband–parent trio approach. Exome data processing, variant calling, and variant annotation were performed by the Chigene Translational Medicine Research Center (Beijing, China) as a clinical service. For whole-exome sequencing (WES), sequencing library was created by Agilent SureSelect Human All Exon V6 Kit (Agilent Technologies, Santa Clara, CA) in accordance with the manufacturer’s protocol. The prepared libraries were sequenced with a HiSeq2500 (Illumina, San Diego, CA). The Genome Analysis Toolkit (GATK) was used for variant calling (GATK HaplotypeCaller) (McKenna et al., 2010). A total of >99% of reads were mapped to genomic targets, with 20× coverage for >95% of bases. In the proband, a total of 118,818 variants were identified in exonic and splice site regions. “Rare deleterious” mutations were defined as those that met the following criteria: 1) their alternative allele frequencies were ≤0.5% in gnomAD database and our local database; 2) the severity of the impact on the protein should be high, such as a stop-gain, stop-loss, nonsense, frameshift, or splice-site mutation. The standard of missense mutation is REVEL ≥0.75 or all the prediction tools score as Damaging; 3) the clinical manifestations of the patient should closely associate with the disease phenotype. All of the mutations were classified following the ACMG/AMP guidelines to define (Richards et al., 2015). We comprehensively evaluated the pathogenicity, frequency, and clinical manifestations of the mutation. Sanger sequencing was used to verify the authenticity of the mutations.

## Sequencing Results

**FIGURE 1:**
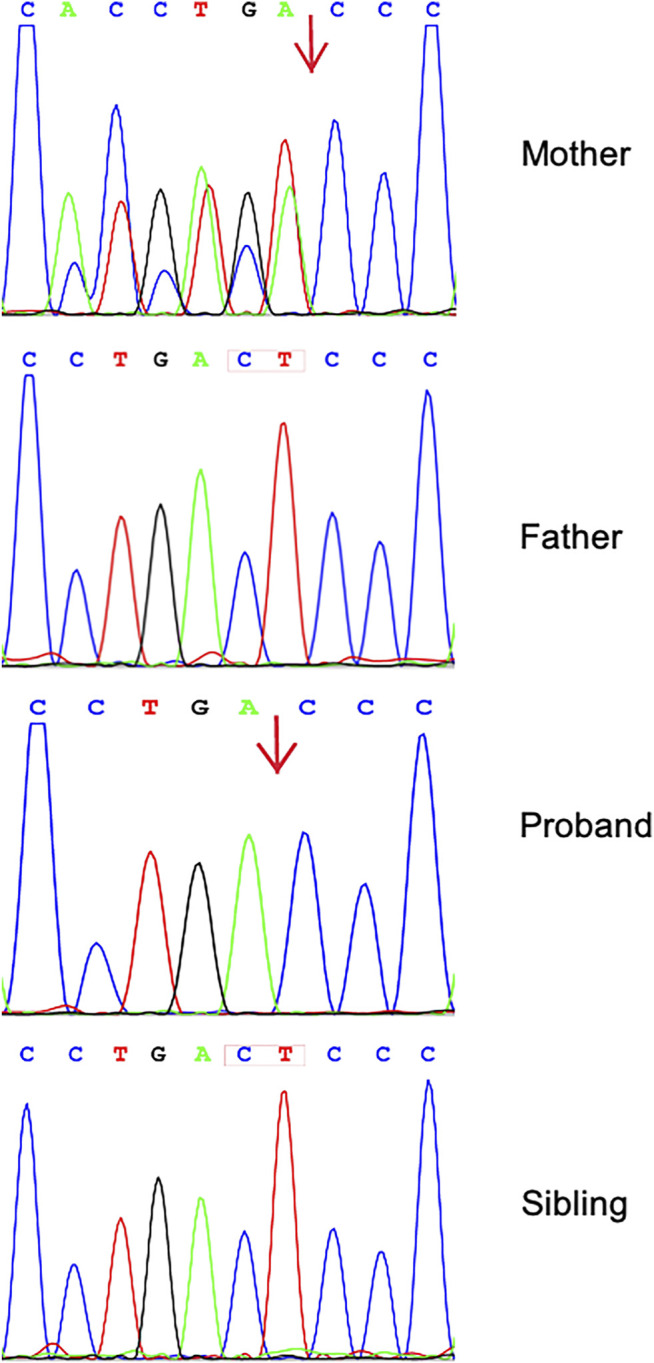
Sequence analysis of *CNTN2* gene from the family. Chromatograms showing that the mother is heterozygous for c.2873_c.2874delCT (p.Thr958Thrfs*17). The proband shows a homozygosity of the mutation; his father and little brother have wild-type sequence.

We identified 11,287 variants existing in genes that matched with known phenotypes. Variants from 1,683 genes associated with epilepsy were subsequently extracted, leading to the identification of a novel homozygous frameshift variant (c.2873_c.2874delCT, p.Thr958Thrfs*17) in the *CNTN2* gene (RefSeq NM_005076) (Supplementary Table 1). Parental WES data and Sanger analysis of the mutation revealed that one copy of the variant was inherited from his unaffected mother; the other variant was not detected in the father’s genome. He has a healthy little brother who carried no variant at this locus (Figure 1).

**FIGURE 2:**
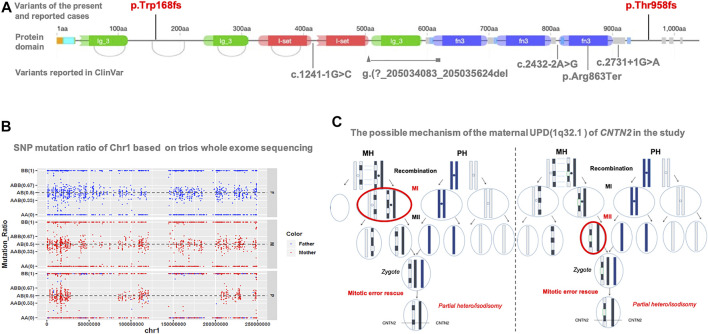
(**A**) Protein structure of CNTN2 and localization of variants reported in the present study, previous literature (red), and ClinVar database (gray). This present variant (red: c.2873_c.2874delCT, p.T958Tfs*17) is in the second last exon but within the last 50aa. There is only one publication (NM_005076.5: c.504del, p.Trp168fs) reported with FAME5. Another five reported loss-of-function variants in ClinVar lack detailed clinical information. The origin graph of protein domain is cited from DECIPHER (https://www.deciphergenomics.org/). **(B)** SNP analysis reveals the altered regions on chromosome 1, including maternal heterodisomy at chr1: 1-26488019, chr1:79411951-150123132, and chr1:206230986-249250621; and maternal isodisomy at chr1: 26488019-49511248, chr1: 50957509-79411951, and chr1: 150123132-206230986 (**C**) Based on the results of SNP analysis, the possible mechanism that results in the mixture pattern of uniparental disomy (UPD) is recombination of maternal homologs (MH) before Meiosis I, then LEFT: chromosome segregation error at Meiosis I (MI), or RIGHT: chromosome segregation error at Meiosis II (MII), and then a rescuing segregation error in the zygote. For simplicity, we only show the chromatid of chromosome pair for UPD with partial hetero/isodisomy. Paternal homologs (PH).

This variant is not in gnomAD. It is located in the second last exon but not within the last 50aa; this frameshift variant is expected to result in a null product due to nonsense-mediated decay. On investigation, only one loss-of-function variant of *CNTN2* has been reported in an individual with a similar presentation as our patient (Stogmann et al., 2013). Five loss-of-function variants (c.1241-1G>C; c.2432-2A>G; g. (?205034083_205035624del; c.2587C>T; c.2731 + 1G > A) are reported in ClinVar as pathogenic or likely pathogenic (Figure 2A). In the HGMD database, six heterozygous variants including four missense, one splice, and one nonsense variant are recorded. The nonsense variant (p.Tyr585Ter) was reported to be associated with autosomal recessive intellectual disability (Harripaul et al., 2018). All other variants were not reported to be associated with a specific phenotype.

The frameshift variant detected in our patient can be classified as pathogenic following the ACMG/AMG guideline (PVS1, PM2_P, PM3_P). Given the atypical inheritance pattern in this case, possibilities including maternal UPD or a paternal deletion including the *CNTN2* locus (at least exon 22) were examined. WES-based copy number analysis of the father’s genomic DNA yielded normal results and a further qPCR detecting genomic copy number abnormalities of exon 22 in *CNTN2* from his father.

As expected, SNP mutation ratio of Chr1 based on trios-WES showed the maternal UPD of chromosome 1 [UPD (1)mat] in the proband. It revealed the altered regions on chromosome one; maternal heterodisomy at chr1:1-26488019, chr1:79411951-150123132, and chr1:206230986-249250621; and maternal isodisomy at chr1: 26488019-49511248, chr1: 50957509-79411951, and chr1: 150123132-206230986 (Figure 2B). *CNTN2* is located on chromosome 1q32.1, from chr1: 205042937 to 205078289, exactly where the isodisomy of chr1: 150123132-206230986 occurred.

## Follow-Up

**FIGURE 3:**
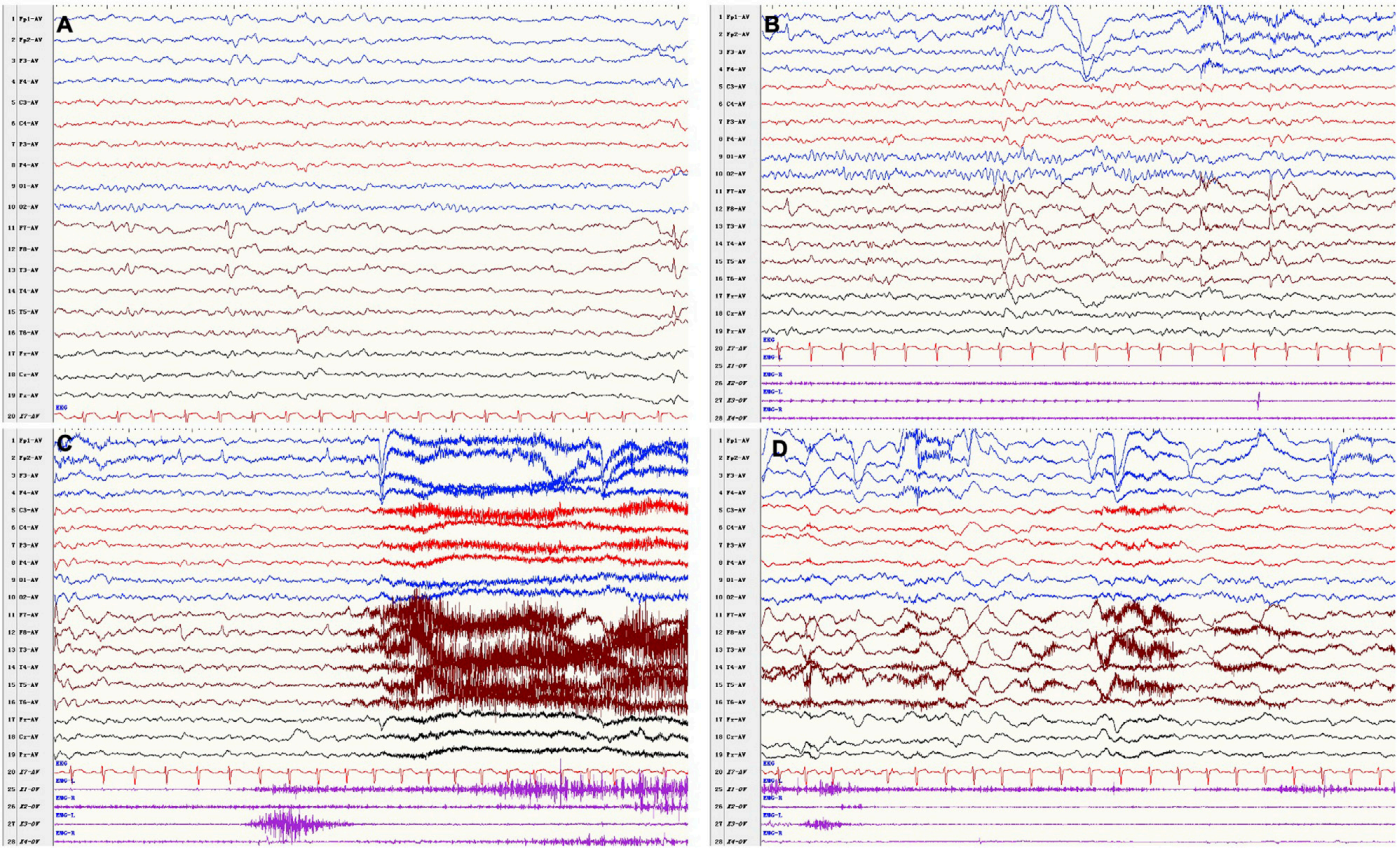
VEEG after drug withdrawal at 10 years 8 months. Interictal **(A,B)**: bilateral frontal–temporal epileptiform discharges, **(B)** was the background a few seconds before the onset. Ictal **(C,D)** recorded a focal seizure (fear, closed eyes, vocalization, forward and backward movements of the hips, pedaling movements of lower limbs) in awake period.

At the follow-up of 9 years old, he had been seizure free for 26 months since add-on therapy of VPA (9.3 mg/kg/day) with OXC remaining at 16.7 mg/kg/day, and LEV had been withdrawn 18 months earlier. The parents of the patient complained that the attack of behavior arrest occurred occasionally, which lasted for less than 10 s. However, EEG failed to monitor the ictal epileptiform discharges. By the last visit, the boy was 10 years 8 months; he had seizure relapse when his parents were trying to withdraw VPA and OXC. The seizure type and ictal EEG were similar as before (Figure 3), and a recurrent brain MRI did not show any abnormality. His seizure was under control with sufficient dose of VPA and OXC. He remained to have cognitive and behavioral deficits, as well as emotional regulation problem. He has normal growth.

## Discussion

We reported a non-consanguineous Han male with homozygous c.2873_c.2874delCT, p.T958Tfs*17 mutation in *CNTN2*, onset with generalized seizure and evolved into focal seizure (Table 1). At present, there was only one family reported worldwide with mutations in *CNTN2* and similar clinical presentation (Stogmann et al., 2013). According to the evidence accumulated from public databases such as HGMD, gnomAD, and ClinVar (Figure 2A), all the variants classified as pathogenic/likely pathogenic belong to loss-of-function.

**TABLE 1:** Summary of *CNTN2* mutations and main clinical features.

	Stogmann et al. (2013)					This report
Individual (sex)	1 (M)	2 (F)	3 (F)	4 (F)	5 (F)	6 (M)
Mutation (NM_005076.5)	p.Trp168fs					p.Thr958Thrfs*17
Age at seizure onset (years)	11	14	11	12	11	6
Age at follow-up (years)	39	37	29	24	21	10.7 (age at last follow-up)
Seizure type	GTCS, CPS	GTCS, CPS	GTCS, CPS	GTCS, CPS	GTCS, CPS	GTCS, CPS
Aura	—	—	—	—	—	−
Cortical tremor	+	+	+	+	+	−
Myoclonic jerks	−	+	+	+	−	−
Cognitive function	NA	IQ 85	IQ 86	IQ 79	IQ 78	Learning difficulties, memory deficits, ADHD traits
Mental disorder	—	—	—	Depressive symptoms	Depressive symptoms	Aggressive, impulsive
Cranial MRI/CT	Normal	Normal	Normal	Normal	MRI bilateral mS	Normal
EEG	—	Temporal IEDs	Normal	Tempoparietal IEDs	Temporal IEDs	T-F IEDs; ictal: T, F onset
AEDs	CBZ	CBZ	CBZ	CBZ, LTG	CBZ	VPA, OXC
Outcomes of AEDs	Good	Good	Good	Poor	Good	Good

M, male; F, female; GTCS, generalized tonic-clonic seizure; CPS, complex partial seizure; IQ, intelligence quotient; OXC, oxcarbazepine; VPA, valproate; LTG, lamotrigine; NA, not available; IED, interictal epileptic discharge; T, temporal; F, frontal; ADHD, attention-deficit hyperactivity disorder; mS, mesial sclerosis.

This is a potentially important case for upgrading the gene–disorder relationship for this emerging seizure disorder. Similar to those patients reported by Stogmann et al. (2013), the patient showed focal seizures, which was indicative of a temporal origin in EEG, and cranial MRI revealing no abnormality. In addition, almost all patients demonstrated a non-progressive course on anti-epileptic therapy.

However, unlike previous patients with familial cortical myoclonic tremor with epilepsy (FCMTE) (Striano and Zara, 2016), the manifestation of myoclonus was not observed in our patient throughout the course of disease. It is unclear whether this symptom was an aspect of *CNTN2*-associated disorder or not.

Furthermore, by summarizing the clinical manifestation of the patients with homozygous mutation in *CNTN2*, we found that focal epilepsy is the main clinical feature, in conjunction with other neuropsychiatric symptoms, whereas our patient displayed more apparent cognitive impairment and emotional regulation problem than previous patients with FCMTE.

Evidence from Harripaul et al.’s study provide a suggestion that loss-of-function variants in *CNTN2* might be responsible for the neuropsychiatric symptoms. In that study, a nonsense variant (p.Tyr585Ter) of *CNTN2* was detected, although it was only supplemented as a candidate gene of autosomal recessive intellectual disability (Harripaul et al., 2018).

Rather than the only explanation of FCMTE, this spectrum of symptoms is more likely to be a consequence of clinical or genetic heterogeneity of *CNTN2*-associated disorder. Due to the limited number of such a rare disease, more cases will help to better understand the phenotype–genotype correlation.

The special autosomal recessive (AR) inheritance presenting in our case was finally confirmed to be a UPD (1)mat of *CNTN2* on 1q32.1. UPD is the presence of a chromosome pair derived only from one parent in a disomic cell line. So far, three types of UPD including isodisomy (UPiD, both copies of a single chromosome homolog are inherited from one parent), heterodisomy (UPhD, a pair of homologous chromosomes are inherited from one parent), and partial heterodisomy/partial isodisomy (segments of both isodisomy and heterodisomy are identified across the chromosome) have been reported (Benn, 2021).

UPD is rare in health populations. In fact, it could become a pathogenic event, when UPD involves imprinted chromosomal region or gene (chromosomes 6, 7, 11, 14, 15, or 20) (Eggermann, 2020), or results in the homozygosity of recessive mutation (Yamazawa et al., 2010). This occurs as a consequence of isodisomy, when one of the parents is a carrier for recessive disorder (Yamazawa et al., 2010).

Just in the case of our study, a mixture of partial heterodisomy and partial isodisomy was identified across the maternal chromosome one from the patient (Figure 2B). The homozygous state of *CNTN2* mutation was the result of maternal UPiD (1q32.1).

There are many possible mechanisms leading to UPD including meiotic non-disjunction with mitotic correction, gametic complementation, mitotic segregation error, or micronuclei (Conlin et al., 2010; Kearney et al., 2011; Benn P, 2020).

Mechanism for the formation of UPD in our case is supposed to be the initial chromosome segregation error during meiosis and a rescuing segregation error in the zygote (Figure 2C).

The occurrence of UPD in the inheritance of autosomal recessive disorder is not very rare (yet at least uncommon), and related studies have been reported since 1988 (Spence et al., 1988; Yamazawa et al., 2010; Soler-Palacín et al., 2018).

Based on the ChromosOmics Database (Liehr T., 2021), 32 records of maternal UPD (1) have been reported (clinical significance of seven cases are unavailable, one is irrelevant). Of the 24 cases with clinical findings, 20 have relevant phenotype with responsible genes located on the UPD, and almost of them are AR (19/20). The remaining four cases are neurodevelopmental disorders without associated pathogenic genes. Yet similar case of maternal UPD (1) of *CNTN2* has not been reported.

In brief, UPD (1) can cause disease not only through imprinting defects but also through homozygous exposure of recessive genes. For fetus with UPD (1) during pregnancy examinations, the recessive genes mapped on the chromosome should be analyzed with caution, and combined with other important clinical features, appropriate management for the genetic diagnosis and counseling should be made.

In recent years, SNP array is recommended in the detection of UPD; it was proven to play an important role in disclosing the mechanisms of UPD (Conlin et al., 2010; Kearney et al., 2011). With the advance of next-generation sequencing, a late work demonstrated that WES-based copy number analysis could also reveal UPD (Soler-Palacín et al., 2018).

Collectively, our study suggested that loss-of-function variants in *CNTN2* may be responsible for temporal epilepsy accompanied with neurodevelopmental impairment. Reporting of this new case will help to upgrade the gene–disease relationship for this emerging *CNTN2*-related disorder. We also provided clinical experience for additional choice of appropriate AEDs for *CNTN2*-related epilepsy. This case revealed a rare a mixture of maternal isodisomy and heterodisomy of chromosome one that unmasked a heterozygous variant.

## Data Availability

All of the data supporting the findings in this study are available upon reasonable request from the corresponding authors.
